# Drug-Repositioning Approaches Based on Medical and Life Science Databases

**DOI:** 10.3389/fphar.2021.752174

**Published:** 2021-11-01

**Authors:** Yoshito Zamami, Hirofumi Hamano, Takahiro Niimura, Fuka Aizawa, Kenta Yagi, Mitsuhiro Goda, Yuki Izawa-Ishizawa, Keisuke Ishizawa

**Affiliations:** ^1^ Department of Clinical Pharmacology and Therapeutics, Tokushima University Graduate School of Biomedical Sciences, Tokushima, Japan; ^2^ Department of Pharmacy, Tokushima University Hospital, Tokushima, Japan; ^3^ Department of Pharmacy, Okayama University Hospital, Okayama, Japan; ^4^ Clinical Trial Center for Developmental Therapeutics, Tokushima University Hospital, Tokushima, Japan; ^5^ Department of Pharmacology, Tokushima University Graduate School of Biomedical Sciences, Tokushima, Japan

**Keywords:** drug repositioning, database, medical information database, life science databases, existing drugs

## Abstract

Drug repositioning is a drug discovery strategy in which an existing drug is utilized as a therapeutic agent for a different disease. As information regarding the safety, pharmacokinetics, and formulation of existing drugs is already available, the cost and time required for drug development is reduced. Conventional drug repositioning has been dominated by a method involving the search for candidate drugs that act on the target molecules of an organism in a diseased state through basic research. However, recently, information hosted on medical information and life science databases have been used in translational research to bridge the gap between basic research in drug repositioning and clinical application. Here, we review an example of drug repositioning wherein candidate drugs were found and their mechanisms of action against a novel therapeutic target were identified via a basic research method that combines the findings retrieved from various medical and life science databases.

## Introduction

Drug repositioning represents a strategy to discover novel pharmacological effects by using the existing approved drugs that have well-proven pharmacokinetics and safety in humans. The advantage of this strategy is that a few necessary steps in drug development are eliminated, thereby reducing the cost and time required for clinical trials as well as the risk of clinical trial failure due to adverse events (Ashburn and Thor, 2004; Chong and Sullivan, 2007; Zamami et al., 2017). Examples of drug repositioning include thalidomide, once used as a sedative, is now used to treat multiple myeloma, and Viagra, which is currently used as an erectile dysfunctions drug (Shim and Liu, 2014). Lee et al. conducted a large retrospective cohort study of 800,000 subjects and reported that metformin, a drug used to treat diabetes, reduced the incidence of colorectal, liver, and pancreatic cancer (Lee et al., 2011). Furthermore, Chamoto et al. reported that the combination of the immune checkpoint inhibitor nivolumab with bezafibrate, a drug for dyslipidemia treatment, increased the antitumor effect of nivolumab by activating the mitochondria of T cells and enhancing their ability to damage cancer cells (Chamoto et al., 2017). In recent years, drug repositioning has attracted attention as a strategy for developing treatments for rare diseases, addressing unmet medical needs, and managing other diseases for which early treatment is desired (Cao et al., 2020; Grein et al., 2020; Wang et al., 2020; Kalil et al., 2021; Salama et al., 2021).

**FIGURE 1:**
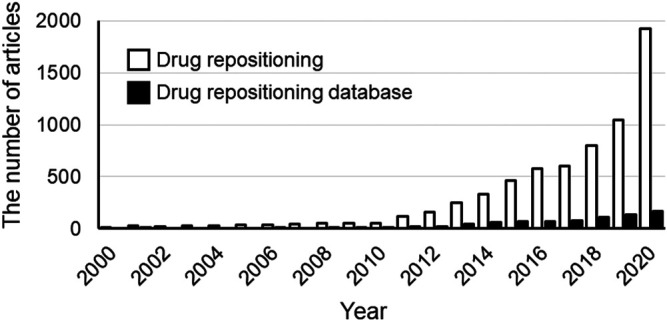
The number of publications from 2000 to 2020. The white and black bars show the results of searches performed in Scopus on the “Drug Repositioning” or “Drug Repositioning” and “databases”, respectively. The number of drug-repositioning studies has increased with the increase in the number of databases. Date of analysis: Jul 9, 2021.

The use of big data has been attracting attention in various fields. In the medical field, databases of medical information, such as prescriptions and databases of spontaneous reports of adverse events caused by drugs and medical devices, are used for research. These databases include data (e.g., prescription data, imaging, clinical data, etc) for patients with various clinical backgrounds [patients (and patient data) who are not always included in clinical trials] and thus enable evaluations that reflect the real-world setting. Therefore, studies to evaluate the efficacy and safety of drugs using medical big data have been actively conducted in many countries worldwide. Drug repositioning is no exception to this trend. The number of papers on drug repositioning is increasing every year, and the number of studies on drug repositioning based on the use of databases is also increasing (Figure 1). The advent of several promising and drug-relevant databases has led to the exploration of a wide range of target coverages and data types (Tanoli et al., 2021). As a gold standard, the repoDB aggregates information on approved and failed drugs (repoDB, 2021). This database can not only provide information on successful drug repositioning to date but also help to identify both true positives (approved drugs) and true negatives (failed drugs), facilitating the computational identification of drug classes considered to have high success rates in drug repositioning (Brown and Patel, 2017).

Databases include life science databases, which host molecular information such as genes and proteins, and medical information databases, which curate clinical information on real-world patients (Table 1). Given the increased interest in drug repositioning, an accumulating body of reviews on the use of databases for drug repositioning has been published (Behl et al., 2021; Kort and Jovinge, 2021; Reay and Cairns, 2021). Several studies reviewing drug discovery approaches using bioinformatics techniques and tools have been reported. Moreover, in silico approaches for drug repositioning in individual diseases such as depression (Mohammad Sadeghi et al., 2021), spinal muscular atrophy (Chong et al., 2021), and colorectal cancer (Beklen et al., 2021) have also been reported. However, many of the approaches used for drug repositioning are based on genome-wide association databases (Behl et al., 2021; Beklen et al., 2021; Chong et al., 2021; Kort and Jovinge, 2021; Mohammad Sadeghi et al., 2021; Reay and Cairns, 2021).

**TABLE 1:** Examples of medical information databases and life science databases.

Type	Characteristics	Name	Website link	References
Spontaneous Adverse Event Reports database	A database that compiles reports of adverse events that occur when a drug is administered to a patient. These databases cataloging the adverse event reports are the most widely used databases for drug repositioning because they can be used to investigate drug-specific adverse events and estimate new drug effects	JADER	https://www.pmda.go.jp/index.html	Pharmaceuticals and Medical Devices Agency, (2021)
		FAERS	https://www.fda.gov/drugs/questions-and-answers-fdas-adverse-event-reporting-system-faers/fda-adverse-event-reporting-system-faers-public-dashboard	FAERS, (2021)
		Vigibase	https://www.who-umc.org/	Uppsala Monitoring Center, (2021)
National sick funds and insurances database	Claims database by National sick funds and insurances is suitable for cohort studies and epidemiological surveys that are conducted over time	NDB	https://www.mhlw.go.jp/stf/seisakunitsuite/bunya/0000177182.html	Ministry of Health Labour and Welfare, (2021)
		JMDC Claims database	https://www.jmdc.co.jp/news/news20200910/	JMDC, (2021)
Gene database	Gene databases can be used to comprehensively examine the genetic information generated by diseases and drugs in the tissues and cells of humans and mice and can be used to elucidate mechanisms of action	GEO	https://www.ncbi.nlm.nih.gov/geo/	NCBI Gene Expression Omnibus, (2021)
		LINCS	https://lincsproject.org/	NIH LINCS PROGRAM, (2021)
		TargetMine	https://targetmine.mizuguchilab.org/ja/	TargetMine, (2021)
		Open Targets Platform	https://platform-docs.opentargets.org/	Open Targets Platform, (2021)
Biobanks	Detailed information on blood, pathology, patient information, etc., can be obtained. Suitable for longitudinal or epidemiological DNA analysis	United Kingdom Biobank	https://www.ukbiobank.ac.uk/	UK Biobank, 2021
		All of Us	https://allofus.nih.gov/funding-and-program-partners/biobank	All of US Research program, (2016)
		China Kadoorie Biobank	https://www.ckbiobank.org/site/	China Kadoorie Biobank, (2021)
		Taiwan Biobank	https://www.twbiobank.org.tw/new_web_en/	Taiwan Biobank, (2019)
		BioBank Japan, National Center Biobank Network, etc.	https://biobankjp.org/https://www.megabank.tohoku.ac.jp/english/sample/https://ncbiobank.org/	Biobank Japan, (2021); Eastern Medical Megabank, (2020); National Center Biobank, (2021)

JADER, Japanese Adverse Drug Event Report database; FAERS, FDA Adverse Events Reporting System; JMDC Claims database, Japan Medical Data Center Claims database; NDB, the National database of Health Insurance Claims and Specific Health Checkups of Japan; GEO, Gene Expression Omnibus; LINCS, Library of Integrated Network-Based Cellular Signatures Data Portal.

On the contrary, medical information databases can be used to examine the efficacy of drugs in actual clinical practice, the occurrence of adverse events, and patient information, such as age and sex. However, a few previous reviews focused on medical databases, and the use of pharmaco-epidemiology approaches to repositioning have been reported. This article describes medical information databases and life science databases that are used by many researchers, including an overview of the latest findings, filling the gap in literature.

## Medical Information Databases

The United States Food and Drug Administration (FDA) (U.S. FOOD and DRUG administration, 2021) Adverse Event Reporting System (FAERS) (FAERS, 2021), the Japanese Adverse Drug Event Report database (JADER) (Pharmaceuticals and Medical Devices Agency, 2021), and the World Health Organization (WHO) database (vigibase) (Uppsala Monitoring Center, 2021) are the main databases—hosting reports associated with spontaneous adverse events—that are assessed in drug-repositioning research. In Japan, the National database of Health Insurance Claims and Specific Health Checkups of Japan (NDB) (Ministry of Health Labour and Welfare, 2021) and the Japan Medical Data Center (JMDC) Claims database (JMDC, 2021) collect the data of insured patients. Of these, vigibase and the JMDC require a certain fee to provide access to the information, whereas databases like FAERS, JADER, and NDB, offer free services.

The US FDA collects spontaneous adverse event reports from medical professionals and companies worldwide. In January 2021, the number of such reports was approximately 12 million. In Japan, the Pharmaceuticals and Medical Devices Agency (PMDA) collects reports regarding spontaneous adverse events and has accumulated approximately 700,000 reports as of January 2021. Furthermore, vigibase, which is curated by the WHO, hosts a large number of reports (∼20 million until 2021); companies from more than 130 countries are involved in vigibase. Vigibase catalogs the data on the time-to-onset (TTO), which is used to investigate the features and the onset of adverse events and is often performed due to the lack of missing data. In recent years, epidemiological studies using databases, such as FAERS, JADER, and vigibase that curate information regarding spontaneous adverse events have been actively conducted to search for drugs with minimal adverse events. Data curation is a key feature that affects the analysis, but until now, individual researchers have used their own curation methods to create datasets. Therefore, unifying such datasets has become a necessary procedure for researchers, especially those who are unfamiliar with the field, to create appropriate datasets, and in recent years, attempts have been made in this direction (Banda et al., 2016).

The NDB collects more than 1.6 billion electronic receipts every year; however, because the NDB is a database that is managed by the government, certain procedures need to be completed before the database can be used for research purposes, which poses a hurdle for new entrants to the receipts research field. The JMDC Claims database hosts data collected from health insurance associations in Japan. It has been collecting data since 2005, with a total of 10 million cases (JMDC 2020 Year News, 2021). The JMDC Claims database contains information on diagnoses of injuries and illnesses, drugs prescribed, and the medical procedures that were performed, and it is possible to evaluate the efficacy and safety of drugs by analyzing data from the JMDC Claims database. Although there is a fee for this service, the database is advantageous in that the procedure is simple, and the data have already been curated to some extent. Moreover, in this database, a unique ID is assigned to each patient, and it is possible to track patients over a long period even if they visit multiple medical institutions or dispensing pharmacies.

## Life Science Databases

Among the life science databases, the Gene Expression Omnibus (GEO) (NCBI Gene Expression Omnibus, 2021), a gene expression database managed by the National Center for Biotechnology Information (NCBI), and the Library of Integrated Network-based Cellular Signatures (LINCS) (NIH LINCS PROGRAM, 2021), a drug discovery tool funded by the National Institutes of Health (NIH), are the most famous. GEO hosts microarray data from more than 4.5 million samples; it is possible to search for experiments of interest (e.g., disease samples or samples of drug-exposed cells) and download the relevant data on GEO. The LINCS program aims to create a database of changes in gene expression and cellular responses to treatment with various compounds. Research is being conducted to develop new therapeutic agents for diseases by comparing the gene expression data associated with the diseases with the LINCS cataloged information on changes in the gene expression using these compounds.

TargetMine (TargetMine, 2021), an integrated data warehousing system developed at the National Institutes of Biomedical Innovation, Health and Nutrition, integrates multiple components, such as genes, proteins, and pathways. Therefore, it is possible to identify related diseases by combining and analyzing data from multiple databases, i.e., specific genes and drugs that target those genes (Chen et al., 2019; TargetMine Data Warehouse for Drug Discovery, 2021)**.** Similarly, the Open Targets Platform (Open Targets Platform, 2021) documents the evidence for the target-disease associations. The platform incorporates molecular data from UniProt, phenotypes from Expression Atlas, and drug information from ChEMBL and is easy to handle (Koscielny et al., 2017).

In recent years, there have been attempts to use biobanks for the transcriptome analysis of biological samples. For example, the UK Biobank (UK Biobank, 2021) is a well-known biobank that curates information regarding blood and urine analysis and the living environment of 500,000 people in the general population. It also stores data genotyping and exome analysis data using genotype arrays from blood DNA (Sudlow et al., 2015). In other countries, research efforts such as the All of US (United States) (All of US Research Program, 2016), China Kadoorie Biobank (China) (China Kadoorie Biobank, 2021), and Taiwan Biobank (Taiwan) (Taiwan Biobank, 2019), are being developed by collecting data from a huge number of cases as part of a national project. In addition, Japan is home to several university hospital biobanks, including the three major biobanks with approximately 700,000 collaborators, i.e., Biobank Japan (Biobank Japan, 2021), Eastern Medical Megabank (Eastern Medical Megabank, 2020), and National Center Biobank (National Center Biobank, 2021). Although it is necessary to go through several contractual processes, such as confidentiality agreements, to handle patient information, it is possible to obtain medical information linked to biological samples, facilitating genomic research that is more in line with clinical practice.

## Use of Pharmacoepidemiology in Drug Repositioning

### Bridging Basic Research to Clinical Research

The prognosis of various diseases, including cancer, has greatly improved with the development of innovative drugs. However, the development cost per drug has been increasing each year worldwide, which could be because clinical trials are becoming increasingly larger. Additionally, the demand for therapeutic agents against intractable and rare diseases is increasing (Dickson and Gagnon, 2004), which has resulted in the emergence of some ultra-expensive drugs (cost of a single treatment ranging from several hundred to several tens of millions of yen). In the conventional drug-discovery model, bridging basic research to clinical research is often a problem, and it is difficult to predict the efficacy and safety of a compound in actual patients based on the results of cell and animal experiments, which decreases the success rate of drug development. Therefore, there is a need to develop therapeutic drugs using unconventional drug discovery models. Drug-repositioning research using medical big data is a good strategy to compensate for the limitations associated with basic pharmacological methods. As existing approved drugs have already undergone clinical trials, various data such as laboratory values and imaging data from each patient are collected over time and accumulated as big data. Research using such medical big data enables the analysis of patients with various backgrounds and in various regions, thus enabling the evaluation of efficacy and safety in actual clinical practice and facilitating the detection of adverse events that are not yet clear among a limited number of patients. Therefore, the drug-repositioning method is considered the best drug-discovery model, which uses big data to bridge basic and clinical medicine.

### Examples of Life Science Database Applications

The technological advancement of omics has generated a large amount of biomedical data, rendering useful resources for drug repositioning. For example, Dudley et al. conducted a study using L1000CDS2—the predecessor of LINCS—and GEO to develop new therapeutic agents for inflammatory bowel disease (Dudley et al., 2011). First, they used microarray data from patients with inflammatory bowel disease downloaded from the GEO database to identify the genes showing alteration in their expression in response to inflammatory bowel disease. Next, these genes were input into LINCS to search for drugs that could counteract the genetic changes caused by inflammatory bowel disease. This resulted in the identification of topiramate, an antiepileptic drug. Subsequently, its efficacy was demonstrated in animal models of inflammatory bowel disease.

### Examples of Medical Information Databases Applications

Several studies have been reported on the use of medical information databases for drug repositioning. For example, Zhao et al. used the medical information databases and the life science database (Zhao et al., 2013). Initially, Zhao et al. used FAERS to identify candidate prophylactic drugs that reduce the incidence of myocardial infarction when used in combination with rosiglitazone and other drugs. In addition, they validated the efficacy of the identified prophylactic candidates using the electronic medical record data of Mount Sinai Medical Center. For the candidate prophylactic agents whose efficacy was confirmed upon the validation of the electronic medical record data, we conducted pathway analysis using the DrugBank and estimated the mechanism of action of the candidate molecules. The presumed mechanisms and the efficacy of the candidate prophylactic agents were verified in animal experiments using mice. The results showed that exenatide, a glucagon-like peptide-1 (GLP-1) receptor agonist, significantly reduced the risk of rosiglitazone-induced myocardial infarction.

In another study, Hashikawa et al. explored the role of the heat shock protein (HSP105) in preventing depression-like behavior in mice (Hashikawa et al., 2017). HSP has neuroprotective effects, and its expression in the brain is known to change under stressful conditions. In this study, assuming an increase in HSP levels in the brain may ameliorate depression, they evaluated the efficacy of teprenone, an HSP inducer, in treating depression. They created a mouse model of depression and measured HSP expression in the brain. The HSP105 expression in the brain was decreased, and the depressive behavior was improved in the teprenone-treated mice. Furthermore, in the JADER database, 1,626 (3.784%) reports of depression in a group of 42,974 individuals without teprenone treatment were identified, wherein only 10 (2.421%) in a group of 413 individuals administered with teprenone showed depression. These data showed a trend toward fewer reports of depression in the teprenone-treated group (odds ratio 0.63; 95% confidence interval [CI] 0.30–1.18; *p* = 0.1920). These results suggest that teprenone may be a novel antidepressant with a different mechanism of action from conventional antidepressants by increasing HSP expression in the brain.

Using the FAERS database, Hamano et al. evaluated the efficacy of diphenhydramine, an antiallergic drug, in preventing cisplatin-induced kidney toxicity (Hamano et al., 2021). They reported that the administration of diphenhydramine in a mouse model of cisplatin-associated renal injury improved the characteristics of renal damage (which included worsening renal function, histopathological changes, elevated inflammatory cytokines, and increased oxidative stress and apoptosis), whereas the histamine knockout mice did not show the same effects. The results suggest that diphenhydramine exerts its effects through mechanisms other than histamine receptors. In addition, they confirmed that diphenhydramine suppressed renal damage without affecting the antitumor effect of cisplatin in mice with embedded cancer. To confirm the efficacy of cisplatin in clinical practice, they conducted a propensity score matching-based retrospective chart review analysis of 1,467 patients who received cisplatin. In 49 matched pairs, diphenhydramine-treated patients had lower incidence of cisplatin-induced renal injury than non-treated patients. Furthermore, the inhibitory effect of diphenhydramine on cisplatin renal injury was seen in all three approaches used in the study, i.e., database study, basic study, and clinical study. Taken together, they proposed diphenhydramine as a candidate drug for prophylaxis against cisplatin-induced renal injury.

### Collaboration Between Life Science and Medical Information Databases

Nagashima et al. focused on finding the molecular mechanism underlying antipsychotic-induced hyperglycemia (Nagashima et al., 2016). First, they searched for drug combinations in the FAERS database and found that a combination with vitamin D analogs significantly decreased quetiapine–induced diabetes mellitus. In clarifying the molecular mechanisms underlying quetiapine-induced hyperglycemia, in GEO, 10 insulin resistance-related genes were found to be downregulated in rat liver after oral quetiapine administration. The most prominent reduction in expression was observed for phosphatidylinositol 3-kinase (PI3K), a known risk factor for diabetes mellitus. Experimental validation using mice revealed that quetiapine induced-insulin resistance was mitigated by cholecalciferol. Further, the PI3K signaling pathway activity was reversed by cholecalciferol supplementation in skeletal muscle, and insulin-stimulated glucose uptake into C2C12 myotube was inhibited in the presence of quetiapine, which was reversed by concomitant calcitriol treatment in a PI3K-dependent manner.

Wakai et al. integrated the in silico and *in vivo* approaches to identify potential therapeutic options for cisplatin-induced renal injury and suggested that this approach could be successful in drug repositioning (Wakai et al., 2020). The study identified a 208-gene expression signature for cisplatin-induced renal injury using the mouse kidney and human kidney organoid transcriptome datasets available in the GEO database. Subsequently, using the bioinformatics database CMap and LINCS Unified Environment (CLUE) and FAERS database, the study identified palonosetron, a serotonin type 3 receptor antagonist, as the candidate drug to reduce cisplatin-induced acute kidney injury. Additionally, clinical data of 103 patients with cisplatin treatment for head and neck cancer demonstrated a higher efficacy of palonosetron in suppressing kidney injury than that of ramosetron. Moreover, the study also showed that the survival rate of zebrafish exposed to cisplatin was significantly increased by palonosetron than by other 5-HT3R antagonists.

**FIGURE 2:**
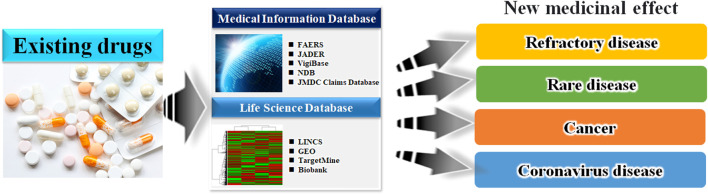
Schematic of the database-based drug repositioning method. By analyzing the database of existing drugs, we can develop novel therapies in areas where there were no treatment options.

**FIGURE 3:**
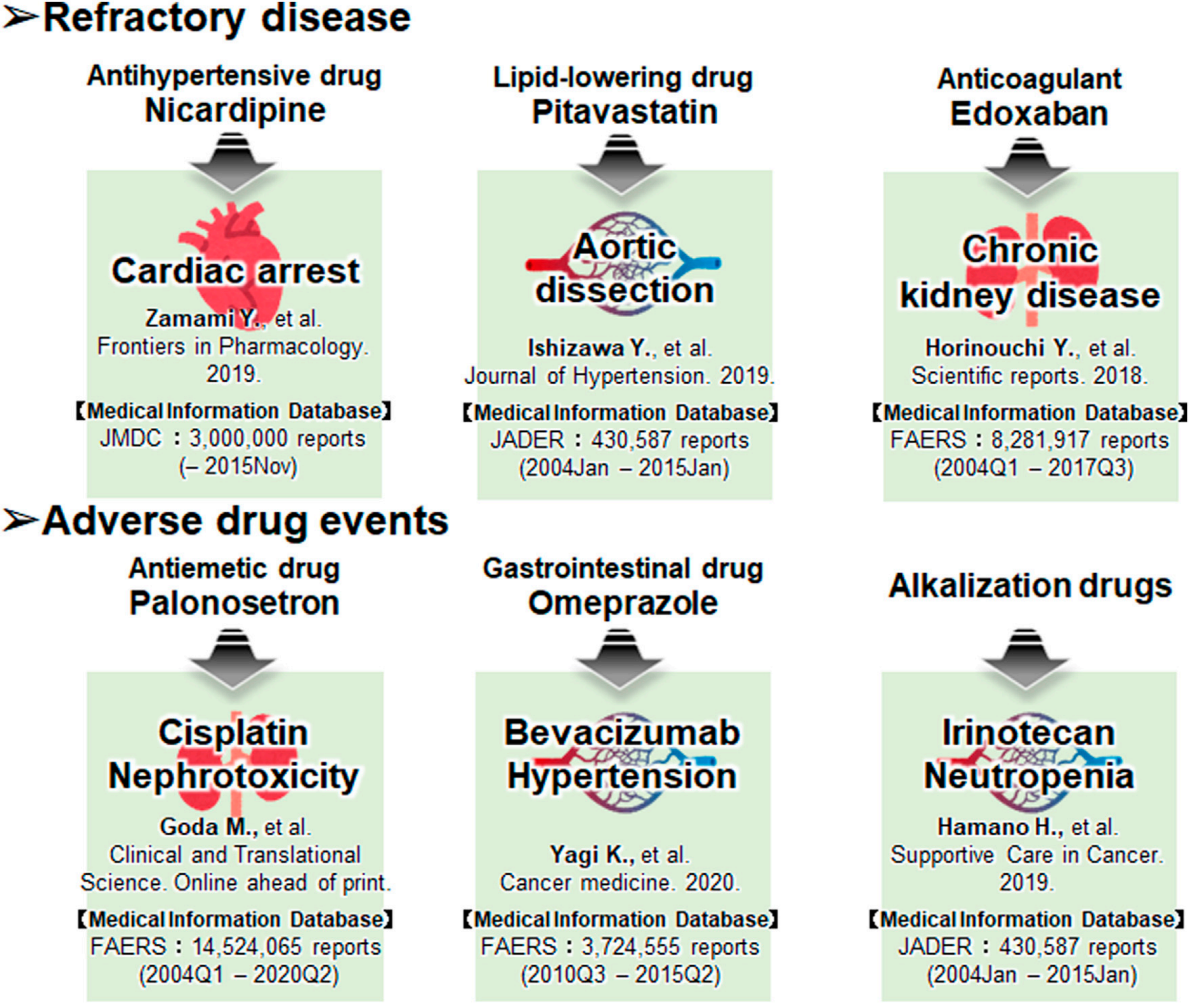
The drug repositioning studies conducted by our research group using the databases. Our future work focuses on developing drugs that have preventive effects against largely intractable diseases and assessing their side effects.

Taken together, it can be inferred that combining the above mentioned medical information data and drug discovery tools could identify more suitable candidates for preventive pharmacotherapy of adverse events and elucidate novel mechanisms of action of the existing approved drugs (Nagashima et al., 2016; Wakai et al., 2020). Moreover, many drug-repositioning studies have been conducted based on information hosted on drug epidemiological databases (Figure 2), indicating the scope of developing novel therapeutic strategies in areas lacking treatment options. In Figure 3, we have summarized the drug repositioning studies conducted by our research group. Additionally, we have planned to identify drugs with preventive effects against largely intractable diseases and assess their side effects of drugs employing data hosted on pharmaco-epidemiological databases (Figure 3).

## Examples of Actual Research: Approaches to Intractable Diseases

We present three examples of drug-repositioning studies based on medical big data for intractable diseases in our field.

The first example is similar to the second one, wherein we combined data searches in the TargetMine with those in the JMDC to extract candidate drugs to improve the prognosis of cardiopulmonary arrest (Zamami et al., 2019). We initially used TargetMine to identify 165 candidate drugs with vasodilatory properties, such as calcium channel blockade; drug data obtained from the JMDC for 2,227 cardiac arrest patients were used to exclude the patient backgrounds based on the propensity score matching, following which, isosorbide nitrate, nitroglycerin, and nicardipine were used as candidate drugs. The adjusted odds ratios for the survival of patients treated with isosorbide nitrate, nitroglycerin, and nicardipine were 3.35, 5.44, and 4.58, respectively. Therefore, the results suggest that isosorbide nitrate, nitroglycerin, and nicardipine may function as new therapeutic agents to improve the prognosis of cardiac arrest patients.

The second example pertains to a study on aortic dissection (Izawa-Ishizawa et al., 2019). Although aortic dissection is a fatal condition, the only effective treatments are surgery and antihypertensive therapy. Using an established mouse model of drug-induced aortic dissection, we investigated the efficacy of pitavastatin, which is known to have endothelial protective effects. Pitavastatin significantly reduced the rate of aortic dissection and rupture in this mouse model. Next, the researchers used JADER to examine the users of 44 drugs at risk for aortic dissection and found that 113 out of 95,090 (0.119%) patients in the non-statin group had an aortic dissection, compared with six out of 16,668 (0.036%) patients in the statin group. Aortic dissection was reported significantly less frequently in the statin group (odds ratio 0.30; 95% CI 0.13–0.69; *p* = 0.0043). The results of basic experiments and the medical information database analysis suggest that statins may inhibit vascular endothelial damage and serve as a new treatment option for aortic dissection.

The third example is of a study on interstitial nephritis (Horinouchi et al., 2018), one of the major causes of chronic kidney disease (CKD) and has emerged as a major clinical issue in recent years. The Factor Xa (FXa) level is increased in various inflammatory diseases; therefore, we investigated the potential application of FXa inhibitors for the treatment of interstitial nephritis. Initially, we examined the effects of FXa inhibitors in a mouse model of renal tubular interstitial fibrosis and found that the FXa inhibitors suppressed fibrosis and inflammatory responses. The FAERS database had 4,895 (0.060%) reports of interstitial nephritis among 8,281,917 cases in the non-FXa inhibitor group) and only 52 (0.039%) reports among 132,591 cases in the FXa inhibitor group (odds ratio 0.65; 95% CI 0.49–0.85). However, for warfarin, both an anticoagulant and an FXa inhibitor, the percentage of interstitial-nephritis-related reports in the total number of reports was 0.060% (4,862/8,131,377) and 0.056% (85/150,540) in the warfarin-free and warfarin-use groups, respectively. There was no significant difference in the frequency of reports of interstitial nephritis between the two groups (odds ratio 0.94; 95% CI 0.76–1.16; *p* = 0.0398). These results suggest that FXa inhibitors may serve as potential preventive agents for CKD that act by inhibiting tubulointerstitial fibrosis.

## Example of Actual Research: Approach to Drug-Induced Adverse Event

Three examples of drug-repositioning studies using medical big data for drug-induced adverse events are presented in this section.

The first approach, similar to the first approach, is for cisplatin renal injury (Goda et al., 2021). Cisplatin is excreted via the multidrug and toxin release (MATE) transporter, and a 5-HT3 receptor antagonist is used as an antiemetic. We investigated the effect of the 5-HT3 receptor antagonist in a mouse model of cisplatin-induced kidney injury and validated it using medical big data. The results of the study were validated using FAERS analysis and a survey of hospital medical records. Concomitant use of first-generation 5-HT3 receptor antagonists (ondansetron, granisetron, and ramosetron) significantly increased the renal accumulation of cisplatin and worsened renal damage, whereas concomitant use of palonosetron had no effect on renal function in the analysis of the data from FAERS and medical records. This revealed that the combination of cisplatin and a first-generation 5-HT3 receptor antagonist was associated with a significant increase in the number of reported adverse renal events compared with the combination of cisplatin and a second-generation 5-HT3 receptor antagonist. These results indicate that second-generation 5-HT3 receptor antagonists can be used safely during cisplatin-induced renal injury therapy.

The second approach was used for bevacizumab-induced hypertension (Yagi et al., 2021). Hypertension is a frequent adverse event in bevacizumab-treated patients, and the development of hypertension has been suggested to be a biomarker of treatment response; FAERS found that hypertension was less frequently reported in bevacizumab-treated patients who were using omeprazole. However, a study of 58 patients diagnosed with colorectal cancer who received initial treatment with XELOX or mFOLFOX6 plus bevacizumab found that patients who used proton pump inhibitors (PPIs) responded more poorly to treatment than those who did not. Using GEO analysis, we estimated the mechanism of action and inferred that PPIs induce vascular endothelial growth factor (VEGF) expression. Using cell lines, we found that PPIs induce *VEGF* expression in colon cancer cells and promote vascular endothelial cell proliferation.

The third approach pertains to a case of prevention of anticancer drug-induced neutropenia (Hamano et al., 2019). Using the data hosted on the JADER database, we reported that alkalinizing agents, such as sodium bicarbonate, reduced the frequency of irinotecan-induced neutropenia. Patients using irinotecan frequently experience adverse events, such as diarrhea and neutropenia. Diarrhea can be alleviated by alkalinizing agents and herbal medicines (Takasuna et al., 1996; Takeda et al., 2001). However, no effective prophylactic agents have been identified for neutropenia to date. One of the causes of neutropenia is SN-38, a metabolite of irinotecan. Alkalinizing agents are known to suppress the reabsorption of SN-38, and we assumed that alkalinizing agents could also suppress irinotecan-induced neutropenia. Therefore, we compared the incidence of neutropenia in patients who received irinotecan with or without alkalinizing agents based on data extracted from electronic medical records. The findings showed that the frequency of neutropenia was low in the patients who used the alkalinizing agent, and the intensity of treatment could be maintained, suggesting that the alkalinizing agent could prolong the survival period. Furthermore, we conducted a more comprehensive analysis using data from the JADER database maintained by the PMDA. We found that the frequency of neutropenia was lower in patients treated with alkalinizing agents in the studies using JADER data.

## Problems Faced by Database Research

In using databases for drug repositioning, one has to be particularly careful about the issue of bias. For example, some of the medical information in the databases we introduced in this article are collected by spontaneous reporting by medical professionals. This leads to many biases, such as inconsistencies in medical terminology due to reporter bias, missing data, multiple reporting, and differences in reporting rates among medical institutions. Therefore, the possibility that the results may include false positives and false negatives as well as true positives must be considered. These demonstrations need to be augmented by approaches other than those involving medical information databases, such as basic pharmacological methods.

On the other hand, the fact that we are not able to make assessments for drug dosage decisions and routes of administration in our study is a current research limitation. Precise dosage determination requires a lot of validation and funding, and in some cases, there is a risk of failure of the clinical trial due to the appearance of adverse events associated with overdose (Peck, 2021). The combination of database analysis methods that can assess this problem is a future challenge.

In addition, retrospective analysis of clinical data from electronic medical records includes noise, and overall data heterogeneity when combining different types of data, such as transcriptome data, chemical structure data, and clinical literature data, poses a challenge for effective drug repositioning. In our research, to take advantage of the characteristics of each, we did not unify them by language or numerical values but suggested multifaceted and robust effectiveness with the final match of each result. However, with future developments, it may be possible to comprehensively analyze clinical data and multiple databases through machine learning methods using natural language. This realization is expected to lead to more consistent and advanced drug discovery strategies.

## Discussion

Numerous medical big data and life science databases have been created. Researchers can choose the database according to the usage situation and the hypothesis they want to prove. A variety of human data on existing approved drugs has been accumulated as big data; therefore, big data are suitable for utilization in drug-repositioning research to identify new applications for the existing approved drugs. Our group is focused on drug-repositioning research to develop preventive drugs for diseases and anticancer drug-induced adverse events using multiple medical big data and life science databases. In these studies, the target population is clearly defined as patients who use drugs, making it easier to conduct both retrospective and prospective studies. Therefore, the level of evidence can be increased by combining the data from these databases with clinical information from facilities with medical information or can undertake collaborative research. Thus, databases are suitable to be used as a bridge between clinical and basic research. By combining medical big data, including actual patient data, with basic pharmacological experimental methods, the limitations of the two methods can be complemented, and the safe and highly effective prophylactic drug candidates can be identified. In addition, life science databases that provide genetic information are compatible with basic research. In the future, it is expected that drug-repositioning research will be actively conducted by utilizing data hosted on these databases and basic research.
